# Relationship between parental psychological control and suicide ideation in Chinese adolescents: Chained mediation through resilience and maladjustment problems

**DOI:** 10.3389/fpsyg.2022.946491

**Published:** 2022-08-18

**Authors:** Ji Sun, Yongfei Ban

**Affiliations:** School of Educational Sciences, Anshun University, Anshun, China

**Keywords:** suicide ideation, parental psychological control, resilience, maladjustment problems, chained mediation model

## Abstract

Suicide ideation is an essential predictor of suicide deaths and is highly prevalent among Chinese adolescents. Several studies have highlighted the significant association between parental psychological control and suicide ideation. However, few studies have focused on the potential mechanisms underlying this relationship. This study investigated the chained mediating effects of resilience and maladjustment problems on the relationship between parental psychological control and suicide ideation among Chinese adolescents. A total of 2,042 students in junior high school completed measurements. The results revealed significant correlations among parental psychological control, resilience, maladjustment problems and suicide ideation. Even after controlling for the effects of gender and grade, parental psychological control positively predicted a significant effect of suicide ideation. Furthermore, suicide ideation was linked with parental psychological control through three pathways: the mediating role of resilience, the mediating role of maladjustment problems, and the chained mediating roles of resilience and maladjustment problems. These findings have broad implications for the field of suicide studies. High levels of parental psychological control, low levels of resilience, and high levels of maladjustment problems may increase the occurrence of suicide ideation.

## Introduction

The latest “Suicide worldwide in 2019” reported that suicide is the fourth leading cause of death among 15–29-year-olds, and more than 7 million individuals die from suicide worldwide every year (World Health Organization [WHO], 2021). For the public and researchers, there has been renewed interest in suicide-related behaviors, especially suicide ideation (SI), which has a high prevalence and is an essential predictor of suicide deaths (Baca-Garcia et al., 2010; Klonsky et al., 2016). SI refers to cognitions that can vary from brief thoughts about death wishes and the worthlessness of life to detailed plans for killing oneself and an intense delusional preoccupation with self-destruction (Sveticic and De Leo, 2012). Previous studies have reported high SI prevalence rates among Chinese children and adolescents (Zhang et al., 2018; Xiao et al., 2019). For example, Zhang et al. (2018) found that the 12-month prevalence of SI was 15.1% among rural Chinese adolescents. Furthermore, SI in adolescents results in subsequent completed suicide and is strongly linked with adverse mental outcomes, such as aggression and depression (Zhang et al., 2018; Xiao et al., 2019). Therefore, it is crucial to understand the potential mechanisms underlying SI among Chinese adolescents. This study focused on the association between parental psychological control (PPC) and SI and clarified the mediating mechanisms underlying this relationship.

As a form of parental control, PPC refers to intruding upon children’s thoughts and feelings in the psychological world and undermining their sense of self (Wang et al., 2007; Li et al., 2016). Compared with parental behavioral control (i.e., the other form of parental control), PPC is the antithesis of autonomy support and an indirect method of influencing children (Baumrind et al., 2010). Several studies have highlighted a significant correlation between PPC and suicide-related behaviors (Ruchkin et al., 2003; Xing et al., 2010; Li et al., 2016). For instance, Xing et al. (2010) found that Chinese adolescents who attempted suicide reported higher levels of psychological control (e.g., perceived parental control). Moreover, some studies have shown that PPC is a more prominent family predictor of adolescent suicidality than parental behavioral control (Li et al., 2016). However, a few scholars have found that the relationship between PPC and SI may be complex (Cero and Sifers, 2013; Li et al., 2016). Specifically, Ruchkin et al. (2003) reported no significant difference in psychological control (e.g., mothers’ rejection and overprotection) between the non-suicidal group and suicidal ideators despite a significant association between SI and perceived negative parental rearing. Furthermore, one study found that SI is unrelated to maternal or paternal control (Wong et al., 2002).

Although the complexity between PPC and SI indicates that the pathways related to these variables vary and different factors might mediate this relationship (Cero and Sifers, 2013; Li et al., 2016), the potential mediating mechanisms remain unclear. However, the influential Double ABCX model could potentially explain how adolescents’ PPC is linked to their SI (McCubbin and Patterson, 1983). As posited by the model, the relationship between the pile-up family stressor *aA* (i.e., PPC) and the negative outcomes *xX* (i.e., SI) is systematically mediated by existing resources *bB* (i.e., resilience) and the individual’s (or family’s) perception of crisis and resources *cC* (i.e., maladjustment problems). Notably, recent studies have demonstrated close relationships among PPC, resilience, maladjustment problems, and SI.

Resilience is commonly defined as the mechanism of adapting well over time in the face of life-changing situations and adverse conditions such as stress and distress (American Psychological Association [APA], 2020). Resilience positively affects the adolescent quality of life and well-being (Migerode et al., 2012). A large body of literature suggests that resilience is significantly associated with PPC (Swanson et al., 2011; Petrowski et al., 2014; Moon et al., 2017). For instance, children with high levels of parental control show low levels of resilience (Swanson et al., 2011). Parental control and overprotection predict resilience levels (Moon et al., 2017).

Conversely, resilience is an essential protective factor against suicide risk (Sher, 2019). And resilience is crucial for reducing SI and suicide attempts (Sher, 2019). Furthermore, resilience might mediate the relationship between PPC and mental health outcomes (Swanson et al., 2011; Moon et al., 2017). Thus, it can be inferred that resilience is crucial in mediating the relationship between PPC and SI.

In adolescents, maladjustment problems (MP) are most involved in internalizing psychological, emotional, and externalizing behavioral problems in various situations (Cui and Conger, 2008; Wang, 2011). Further, MP is a risk factor for suicidal behaviors (Restrepo et al., 2016), and a negative parenting style (e.g., authoritarian corporal punishment) predicted the level of resilience and MP (Eisenberg et al., 2009). Research has also implicated MP as a pathway linking an individual’s negative outcomes, mental resources, and suicidal behaviors (Restrepo et al., 2016). Thus, it is plausible that MP mediates the relationship between PPC, resilience, and SI.

Given the above, we hypothesized that the relationship between PPC and SI might be mediated first by resilience and then by MP. Moreover we inferred that the chained mediating effects of resilience and MP might be related to the relationship between PPC and SI.

The present study examined the potential mechanistic pathways from PPC to SI in Chinese adolescents. Based on the influential double ABCX model and previous studies, we considered the chained mediating effects of resilience and MP on this link. This study hypothesized that: (1) PPC is strongly related to SI among Chinese adolescents, (2) resilience mediates the relationship between PPC and SI, (3) MP mediates the relationship between PPC and SI, and (4) both resilience and MP have a chain mediating effect on the relationship between PPC and SI.

## Materials and methods

### Participants

This study recruited 2,042 students from six junior high schools in Anshun, Zunyi, and Guiyang city in China. A total of 71 participants (3.5%) were excluded from the analyses because they failed to complete the self-report measures. The students’ ages ranged from 13 to 16 years (*M*_*age*_ = 13.91, *SD*_*age*_ = 0.79). Approximately 54.3% of the students were female and 45.7% were male. Of the students, 36.4%, 36.5%, and 27.1% were in seventh, eighth, and ninth grades, respectively.

### Measures

#### Suicide ideation

The 26-item self-report scale evaluates the SI of Chinese adolescents, including the subscales of *despair*, *optimism*, *sleep*, and *lie* (Xia et al., 2002). The scale uses a “yes-no” forced-choice response. Sample items were as follows: “I want to end my life” (*despair*); “I always feel that life is valuable” (*optimism*); “Sometimes I suffer from insomnia due to worry” (*sleep*); and “Sometimes I tell lies” (l*ie*). The sum of despair, optimism, and sleep items was added as the total score. Higher total scores indicated higher levels of SI. The scores of 12 or higher and the sum of lie items lower than four indicate that the participant had SI (Xia et al., 2002).

Previous studies have provided evidence that the scale is a localized tool for accurately evaluating the SI of Chinese undergraduate and junior middle school students (Shi and Xie, 2019; Yuan and Fan, 2021). To further assess the reliability and validity of the scale in adolescents, we recruited 496 junior high school students (girls: *n* = 257, boys: *n* = 239; *M*_age_ = 13.95, *SD*_age_ = 0.79, range_age_ = 12–15 years) and conducted a pilot study. We found internal consistency for overall (α = 0.80), despair (α = 0.78), optimism (α = 0.72), and sleep (α = 0.60). The results suggest that the scale has been validated in a sample of Chinese adolescents. In the present study, the internal consistency coefficient of the scale was 0.83.

#### Parental psychological control

The 18-item children-report scale assesses the PPC of Chinese adolescents and consists of *guilt induction*, *love withdrawal*, and *authority assertion* subscales (Wang et al., 2007). The scale uses a 5-point Likert response scale with scores ranging from 1 (“not at all true”) to 5 (“very true”). Sample items were as follows: “My parents tell me that I should feel guilty when I do not meet their expectations” (*guilt induction*); “My parents act cold and unfriendly if I do something they do not like” (*love withdrawal*); and “My parents tell me that what they want me to do is the best for me, and I should not question it” (*authority assertion*). The mean of 18 items was recorded as the total score, with higher scores indicating higher levels of PPC. In the present study, the internal consistency coefficient of the scale was 0.92.

#### Resilience

A 27-item self-report scale was administered to assess the resilience of Chinese adolescents, including the subscales of *goal focus*, *emotional control*, *positive cognition*, *family support*, and *interpersonal assistance* (Hu and Gan, 2008). The scale uses a Likert response ranging from 1 (“strongly disagree”) to 5 (“strongly agree”). Sample items were as follows: “I have a clear goal in my life” (*goal focus*); “Failure and frustration can make me doubt my ability” (*emotional control*); “I think there is a positive side to everything” (*positive cognition*); “My parents respect my opinion” (*family support*); and “When I am in trouble, I will take the initiative to talk to others” (*interpersonal assistance*). The mean of the 27 items was calculated to obtain a resilience score, with higher scores indicating higher levels of resilience. In the present study, the internal consistency coefficient of the scale was 0.90.

#### Maladjustment problems

The 21-item self-report scale was used to measure the MP of Chinese adolescents, including the subscales of *self-related problems*, *psycho-physiological developmental problems*, *family life basis*, *campus life basis*, and *peer relationship difficulties* (Chen, 2000; Liu and Shu, 2004; Wang, 2011). The scale uses a response ranging from 1 (“almost never”) to 4 (“almost always”). Sample items were as follows: “I do not think I am smart enough” (*self-related problems*), “I will be very angry if someone nicknames me because of my appearance” (*psycho-physiological developmental problems*), “I will be scolded by my parents at home” (*family life basis*), “I think the content of the textbook is too difficult that I do not even understand” (*campus life basis*), and “I do not think my classmates like to play with me” (*peer relationship difficulties*). The higher the mean of all items, the higher the level of MP. In the present study, the scale had an internal consistency coefficient of α = 0.92.

### Data analysis

SPSS Windows software version 27.0 was used to conduct the data analyses. First, Harman’s single-factor test was conducted to assess common method bias (Aguirre-Urreta and Hu, 2019). The results showed that the most significant factor accounted for 22.71% of the variance, and the value was less than the accepted significance cut-off of 50%. This indicated that no significant common method bias was found among any of the variables in this study. Second, the independent sample *t*-test and one-way analysis of variance (ANOVA) were used to examine the differences in gender and grade in the levels of SI. Third, Pearson correlation analyses examined the relationships among all variables. Finally, we used the PROCESS macro in SPSS to examine the chain mediation models of PPC, resilience, and MP on SI. The PROCESS macro uses a path analysis modeling tool based on regression to assess variables’ direct and indirect effects (Hayes, 2018). Here, we performed chained mediation analyses using Model 27 with 5,000 bias-corrected bootstrap samples. The independent variable was PPC (X), the mediating variables were resilience (M_1_) and MP (M_2_), the dependent variable was SI (Y), the control variables were grade and gender. The gender and grade variables were dummy coded (gender: male = 1, female = 0; grade: grade 7 = 0, grade 8 = 1, grade 9 = 2). *a*_1_, *a*_2_, *b*_1_, *b*_2_, and *d*_21_ represent the total effects of X→M_1_, X→M_2_, M_1_→Y, M_2_→Y, and M_1_→M_2_, respectively. The estimated coefficients a_1_ × b_1_, a_2_ × b_2_, and a_1_ × d_21_ × b_2_ provide measures of the indirect effects of X→M_1_→Y, X→M_2_→Y, and X→M_1_→M_2_→Y, respectively. Bias-corrected bootstrap confidence intervals (CIs) were calculated with a yield of 95%. If CI excluded zero, the chained mediation model was significant.

## Results

### Descriptive statistics and correlation analyses

First, the SI rate in this study was 14.3% (*n* = 282), which is consistent with the findings of Zhang et al. (2018). Second, the independent sample *t*-test and ANOVA were used to examine the differences in gender and grade in the levels of SI. The results of the *t*-test showed a significant effect of gender on the level of SI, *t*_(1969)_ = 7.13, *p* < 0.001. The female students (*M* = 6.10, *SD* = 5.27) reported higher levels of SI than their male counterparts (*M* = 4.51, *SD* = 4.48). The results of the ANOVA indicated a significant grade difference in SI, *F*_(2,1968_*_)_* = 7.24, *p* = 0.001. Students in grade 7 (*M* = 4.81, *SD* = 4.83) reported a significantly lower level of SI than students in grades 8 (*M* = 5.69, *SD* = 5.06) and 9 (*M* = 5.70, *SD* = 5.04).

Descriptive statistics and Pearson correlations for all variables are presented in Table 1. Pearson correlation analysis was used to test the relationships between all variables. The results showed that SI was significantly correlated with PPC (*r* = 0.39, *p* < 0.001), resilience (*r* = –0.67, *p* < 0.001), and MP (*r* = 0.70, *p* < 0.001). This suggests that a high level of SI is associated with a high level of PPC, a low level of resilience, and a high level of MP. PPC was significantly correlated with resilience (*r* = –0.41, *p* < 0.001) and MP (*r* = 0.52, *p* < 0.001), indicating that participants who reported high levels of PPC also reported low levels of resilience and high levels of MP. A negative correlation was found between resilience and MP (*r* = –0.67, *p* < 0.001). This implies that a high level of resilience is associated with a low level of MP.

**TABLE 1:** Descriptive statistics and Pearson correlations matrix (*n* = 1971).

	M	SD	1	2	3	4
1. Parental psychological control	2.39	0.73	1			
2. Resilience	3.38	0.59	–0.41***	1		
3. Maladjustment problems	2.04	0.53	0.52***	–0.67***	1	
4. Suicide ideation	5.38	4.99	0.39***	–0.67***	0.70***	1

M = mean, SD = standard deviation.

*** p < 0.001.

Moreover, we performed linear regression analysis with PPC as a predictor and SI as the dependent variable. Gender and grade were used as control variables. As shown in Table 2, even after controlling for the effects of gender and grade, PPC still predicted a significant effect of SI (β = 0.40, *p* < 0.001).

**TABLE 2:** Multiple linear regression results for testing parental psychological control (PPC) in predicting suicide ideation (SI) (*n* = 1971).

	Model 1			Model 2		
		
	B	β	t	B	β	t
**Control variable**						
Gender	–1.57	–0.16	–7.04***	–1.64	–0.17	–8.04***
Grade	0.44	0.07	3.13***	0.48	0.08	3.75***
**Predictor variable**						
Parental psychological control				2.71	0.40	19.48***
R^2^	0.03			0.19		
F	30.39***			150.58***		
ΔR^2^				0.16		
ΔF				379.29***		

Grade: 2 = grade 9, 1 = grade 8 and 0 = grade 7. Gender: 1 = male and 0 = female.

*** p < 0.001.

### The chained mediation analyses

Three models were used to assess the chained mediation of resilience and MP in the relationship between PPC and SI after controlling for the effects of grade and gender (see Table 3). We found that PPC had a significant negative effect on resilience (β = –0.43, *p* < 0.001) in model 1 and a significant positive effect on MP (β = 0.32, *p* < 0.001) in model 2. Importantly, resilience had a direct negative predictive effect on MP (β = –0.52, *p* < 0.001) in model 2. Additionally, resilience (β = –0.36, *p* < 0.001) and MP (β = 0.44, *p* < 0.001) significantly predicted the level of SI in model 3. Nevertheless, PPC had no significant effect on SI (β = 0.02, *p* = 0.40).

**TABLE 3:** The chained mediation models of resilience and maladjustment problems (MP) in the relationship between parental psychological control (PPC) and suicide ideation (SI) after controlling for the effects of gender and grade (*n* = 1971).

Predictor variable	Outcome variable	R	R^2^	F	β	t	Boot LLCI	Boot ULCI
**Model 1**								
Parental psychological control	Resilience	0.43	0.18	147.28***	–0.43	–20.22***	–0.47	–0.38
Gender					0.23	5.60***	0.15	0.31
Grade					–0.07	–2.55	–0.11	–0.02
**Model 2**								
Parental psychological control	Maladjustment problems	0.75	0.56	619.15***	0.32	18.72***	0.28	0.36
Resilience					–0.52	–31.39***	–0.55	–0.48
Gender					–0.25	–8.57***	–0.31	–0.20
Grade					0.18	9.87***	0.15	0.22
**Model 3**								
Parental psychological control	Suicide ideation	0.75	0.56	501.13***	0.02	0.85	–0.02	0.05
Resilience					–0.36	–17.86***	–0.40	–0.32
Maladjustment problems					0.44	19.70***	0.40	0.49
Gender					–0.08	–2.51*	–0.13	–0.02
Grade					–0.03	–1.31	–0.06	0.01

Grade: 2 = grade 9, 1 = grade 8 and 0 = grade 7. Gender: 1 = male and 0 = female.

* p < 0.05, *** p < 0.001.

**FIGURE 1:**
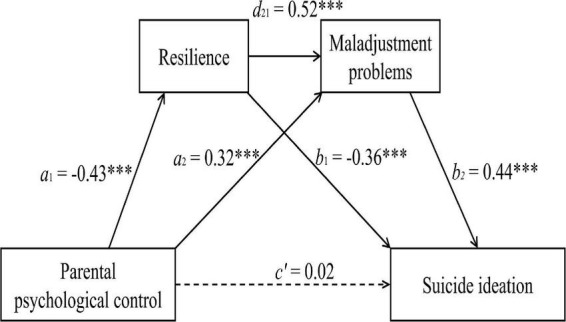
The chained mediation model (*n* = 1971). ^***^p < 0.001.

The results of the chained mediating roles of resilience and MP after controlling for the effects of grade and gender are displayed in Figure 1 and Table 4. These findings demonstrated that the total indirect effect (including three different pathways) was 0.39 (95% CI: [0.36, 0.43]), which accounted for the 96.1% of the total effect. Specifically, the indirect effects 1, 2, and 3 linked PPC with SI via resilience, via MP, and serially via resilience and MP, respectively. Moreover, the indirect effects 1, 2, and 3 explained the 37.6% (95% CI: [0.13, 0.18]), 34.4% (95% CI: [0.12, 0.16]), and 24.1% (95% CI: [0.08, 0.12]) of the total effect. None of the 95% CIs overlapped with zero, indicating that all indirect effects were statistically significant.

**TABLE 4:** The direct and indirect effects in chained mediation model after controlling the effects of gender and grade (*n* = 1971).

	Effect	BootSE	Boot LLCI	Boot ULCI	Ratio of indirect to total effect
Total effect	0.41	0.02	0.37	0.45	—
Direct effect	0.02	0.02	–0.02	0.05	—
Total indirect effect	0.39	0.02	0.36	0.43	96.1%
Indirect effect 1	0.15	0.01	0.13	0.18	37.6%
Indirect effect 2	0.14	0.01	0.12	0.16	34.4%
Indirect effect 3	0.10	0.01	0.08	0.12	24.1%

Indirect effect 1: PPC → resilience → SI. Indirect effect 2: PPC → MP → SI. Indirect effect 3: PPC → resilience → MP → SI. Boot SE, Boot LLCI and Boot ULCL were estimated standard error, 95% confidence interval lower and 95% confidence interval upper.

## Discussion

The present study investigated the relationship between PPC and SI and the crucial chained mediating roles of resilience and MP in this relationship in a sample of Chinese adolescents. More importantly, the results revealed that the strong relationship between PPC and SI might be due to resilience and MP.

The results revealed gender-based differences in SI levels. Female students had a significantly higher SI than male students. This finding is consistent with that of Xiao et al. (2019). Previous studies have also suggested that one of the crucial risk factors for the incidence of suicide is the female sex (Kidd et al., 2006; Ballester et al., 2021). Additionally, statistical grade differences were observed at the SI level. Grade 8 and 9 students reported higher levels of SI than grade 7 students. In China, students take a senior high school entrance examination at the end of Grade 9. Thus, students in grades 8 and 9 might suffer more stress, which might be linked to psychological and behavioral problems. Our results revealed that the students in grades 8 (*M* = 2.07, *SD* = 0.51) and 9 (*M* = 2.16, *SD* = 0.51) had significantly higher scores for MP than students in grade 7 (*M* = 1.93, *SD* = 0.53; *F_(2,1968)_* = 30.82, *p* < 0.001). Given these differences in gender and grade, it is necessary to control for the effects of these variables when testing the associations of SI with other variables.

In the present study, the correlation analyses showed that PPC was significantly associated with SI even after controlling for the effects of gender and grade, suggesting that PPC is an essential predictor of SI. This result is consistent with previous studies linking PPC to SI (Ruchkin et al., 2003; Li et al., 2016).

Notably, the mediation analyses showed that PPC was indirectly linked to SI via three pathways: resilience, MP, and the chained mediating effects of resilience and MP. First, resilience is a crucial mediating factor in the relationship between PPC and SI. Specifically, students with higher levels of PPC tended to have lower levels of resilience, leading to higher levels of SI. This finding is partly in line with previous studies that suggested that resilience plays an essential mediating role in the relationship between parental rearing behavior (e.g., parental control) and negative mental health problems (Petrowski et al., 2014; Moon et al., 2017). Second, MP has a mediating effect on the relationship between PPC and SI. Students with higher levels of PPC tended to have higher levels of MP, which increased the incidence of SI. This finding is not surprising given that previous research has found that MP is indicated by the parental rearing style (Eisenberg et al., 2009) and is a significant predictor of suicidal behaviors (Restrepo et al., 2016). Finally, and most importantly, a significantly chained mediation model of PPC → resilience → MP → SI was found. This illustrates that resilience mediated the relationship between PPC and MP, whereas MP played a mediating role in the relationship between resilience and SI. Specifically, individuals with higher levels of PPC reported lower levels of resilience, which may contribute to higher levels of MP and lead to higher levels of SI. A series of studies have shown that individuals with higher levels of PPC would tend to have lower levels of resilience (Anwer et al., 2019), and lower levels of which might be associated with higher levels of MP (Eisenberg et al., 2009; Zhuo et al., 2021). Moreover, the higher levels of MP with a higher possibility of SI were in line with the findings that individuals with higher levels of MP reported higher levels of suicide (or death) ideation (Smith et al., 2012; Iranzo et al., 2019).

The findings of this study have several important implications for the field of suicide studies. First, the chained mediation model deepens our understanding of the mechanism of the relationship between PPC and SI. This demonstrated that PPC is related to SI through resilience and MP. These findings provided insights into the inconsistent results of PPC on SI, and the indirect pathways between PPC and SI may explain these inconsistencies. Second, our findings have important implications for intervention. This implies that interventions for adolescents with high levels of SI might focus on reducing PPC, increasing resilience, and reducing MP. In collectivist cultures, parental control (including psychological and behavioral control) is considered as an essential responsibility and is frequently used by parents in different situations (Shek, 2007; Li et al., 2015). Given the complexity of changing parental rearing methods and reducing internalizing and externalizing problems for adolescents, interventions for adolescents with SI may be more effective when they have a strong focus on resilience.

Although the present study has some strengths, several limitations must be acknowledged, and our results must be interpreted cautiously. First, this study’s cross-sectional design did not allow us to infer causality associations. It was challenging to examine the prospective and longitudinal changes in PPC, resilience, MP, SI, and their relationships. Second, the focus of this study was limited to PPC. Some studies have suggested that other family factors, such as parental warmth (Li et al., 2016) and family socioeconomic status (Raschke et al., 2022), might be strongly associated with suicide risk. Future studies should also consider these variables. Third, the mediating effect of resilience and MP accounted for approximately 96.1% of the total effect in the present study. However, it is also vital for future research to explore other mediators, such as hopelessness (Li et al., 2016), and investigate protective factors (e.g., Perceived social support; Xiao et al., 2020) for SI.

## Conclusion

In summary, this study investigated the mechanisms involved in the relationship between PPC and SI. The results suggest a chained mediating role of resilience and MP in PPC and SI relationships. These findings can guide the direction of interventions that reduce the high rate of SI among adolescents. In particular, improving resilience and reducing MP may effectively reduce suicide risk.

## Data availability statement

The data presented in this study are available on request from the corresponding author. The data are not publicly available due to the privacy of the participants.

## Ethics statement

The studies involving human participants were reviewed and approved by School of Educational Sciences, Anshun University, China. Written informed consent to participate in this study was provided by the participants’ legal guardian/next of kin.

## Author contributions

JS designed the study, collected the data, analyzed the data, and wrote the manuscript. YB collected the data, analyzed the data, and reviewed the manuscript. Both authors contributed to the article and approved the submitted version.
